# How CAGE, RAPS4-QF, and AUDIT Can Help Practitioners for Patients Admitted with Acute Alcohol Intoxication in Emergency Departments?

**DOI:** 10.3389/fpsyt.2014.00072

**Published:** 2014-06-24

**Authors:** Georges Brousse, Benjamin Arnaud, Julie Geneste, Bruno Pereira, Ingrid De Chazeron, Frederique Teissedre, Christophe Perrier, Raymund Schwan, Laurent Malet, Jeannot Schmidt, Pierre Michel Llorca, Cheryl J. Cherpitel

**Affiliations:** ^1^Service Psychiatrie et Addictologie de l’Adulte CMP B, CHU Clermont Ferrand, Clermont Ferrand, France; ^2^EA 7280 UFR Médecine, Université Clermont 1, Clermont Ferrand, France; ^3^Service Accueil Urgences, CHU Clermont Ferrand, Clermont Ferrand, France; ^4^Delegation Recherche Clinique et Innovation, CHU Clermont Ferrand, Clermont Ferrand, France; ^5^Laboratoire de Psychologie Sociale et Cognitive (LAPSCO), Université Blaise Pascal, Clermont Ferrand, France; ^6^Service de Psychiatrie, CHU Nancy, Toul, France; ^7^Alcohol Research Group, Emeryville, CA, USA

**Keywords:** drunkenness, CAGE, AUDIT, RAPS4-QF, brief interventions, emergency department

## Abstract

**Aims:** To help clinicians to identify the severity of alcohol use disorders (AUDs) from optimal thresholds found for recommended scales. Especially, taking account of the high prevalence of alcohol dependence among patients admitted to the emergency department (ED) for acute alcohol intoxication (AAI), we propose to define thresholds of severity of dependence based on the AUDIT score.

**Methods:** All patients admitted to the ED with AAI (blood alcohol level >0.8 g/L), in a 2-month period, were assessed using the CAGE, RAPS-QF, and AUDIT, with the alcohol dependence/abuse section of the mini international neuropsychiatric interview (MINI) used as the gold standard. To explore the relation between the AUDIT and the MINI the sum of the positive items on the MINI (dependence) as a quantitative variable and as an ordinal parameter were analyzed. From the threshold score found for each scale we proposed intervals of severity of AUDs.

**Results:** The mean age of the sample (122 males, 42 females) was 46 years. Approximately 12% of the patients were identified with alcohol abuse and 78% with dependence (DSM-IV). Cut points were determined for the AUDIT in order to distinguish mild and moderate dependence from severe dependence. A strategy of intervention based on levels of severity of AUD was proposed.

**Conclusion:** Different thresholds proposed for the CAGE, RAPS4-QF, and AUDIT could be used to guide the choice of intervention for a patient: brief intervention, brief negotiation interviewing, or longer more intensive motivational intervention.

## Introduction

Emergency departments (EDs) are special places for primary care, where frequent heavy and problem drinking are prominent among admissions (1–5). Identification of patients with alcohol use disorders (AUDs) is important in EDs, since these patients may be motivated to change drinking behaviors and to accept treatment or referral for problem drinking, particularly if their admission was related to their alcohol use (6). Indeed, in EDs, patients with alcohol-related disorders may be more prone to initiating a course of treatment following intervention by a health professional who can demonstrate their alcohol abuse and suggest an appropriate therapeutic strategy (7, 8). The optimal aim of alcohol interventions in ED is to assist the patient in reducing consumption (in the case of risky drinking and abuse) or to enter into specialized alcohol treatment for those who are alcohol dependent (9–11). Different types of Interventions [brief alcohol interventions (BAIs), brief negotiation interviewing (BNI), or motivational intervention (MI)], which require different type of people (ED workers, ED workers trained to alcohol interventions, or trained alcohol health workers) can be offered.

Brief alcohol interventions have been found to be effective in a number of clinical settings in primary care practice (12–14). They are comprised of short counseling sessions, which can be provided by emergency staff (nurses, physicians) and have a very good cost-time-effectiveness ratio for those with unhealthy alcohol use (15). However, there is little evidence for efficacy of BAIs among patients with very heavy alcohol use or dependence, particularly those identified by screening tests who are not actively seeking help or advice and who therefore are less likely to be amenable to change (16). Particularly, a majority of patients admitted to the ED for acute alcohol intoxication (AAI) fit this profile (17). In order to assist these patients in changing drinking behavior, BAIs can incorporate BNI in time limited health care settings (9, 18). BNI can be provided by ED workers trained to alcohol interventions or ideally trained alcohol health workers who are aware of the psychopathologic underpinnings of dependence and are able to address the patient’s ambivalence and perceived difficulty in behavioral change related to his alcohol problem (19). Nevertheless, BNI interventions may still result in failure (16). Thus, severely dependent patients who often exhibit significant denial of their alcohol problems (20) may require a more lengthy MI (60 min compared to the usual 15 or 20 min), which may be more effective in addressing excessive resistance (21). MI requires a high level of understanding of the psychological mechanisms of dependence and must be understood as adopting an empathic and non-confrontational style. This psychological intervention (MI) is not classically a part of the ED culture where important barriers to translating alcohol interventions to clinical practice exist, and requires participation of trained alcohol health workers (22).

To maximize their interest, these different types of Interventions, which require different type of people (ED workers, ED workers trained to alcohol interventions, or trained alcohol health workers) should be adjusted with severity of Alcohol Use Disorder (AUD). It is therefore important to identify as precisely as possible the severity of the drinking problem (at-risk, abuse, dependence, severe dependence) by using screening tools capable of distinguishing these conditions, so that each patient may be offered the most suitable intervention. Thus, ED admission for drunkenness is an opportune time for initiating interventions for AUDs but the specific screening test used to identify severity of the disorder and the resulting intervention must be determined (23, 24). Hungerford and Pollock have recommended that screening instruments have high sensitivity and specificity, and are not time consuming, expensive, or difficult to use (25). The use of the CAGE (26–28), RAPS4-QF (29), and AUDIT (30–32) tests have all been recognized as effective for detecting alcohol-related disorders in the ED setting (1, 2, 4, 33, 34). Comparisons, taking into account gender, of the performance of these screening instruments among intoxicated patients in a French emergency service site have been reported, which included the optimal cut points for detecting different levels of severity of AUDs for each of these scales (35). As reported in the earlier paper, for detecting alcohol abuse the optimal cut point for the RAPS4-QF was ≥2 for men and ≥4 for women. In the case of CAGE, the optimal cut point was ≥3 for men and ≥2 for women. The optimal cut point for the AUDIT was ≥12 for men and ≥7 for women. For detecting alcohol dependence, the optimal cut point for the RAPS4-QF was ≥3 for men and ≥4 for women. For the CAGE, a cut point ≥3 was found for both men and women. For AUDIT, an optimal cut point ≥18 was found for the total sample, ≥14 for men and ≥11 for women (35). However, to our knowledge, there are no studies, which propose to graduate levels of severity of dependence (moderate or severe) by these screening scales in ED for patient admitted for IAA.

The aim of this paper was to help clinicians to identify the severity of AUD from optimal thresholds found for recommended scales. Especially, taking account of the high prevalence of alcohol dependence among patients admitted to the ED for AAI, we have tried to define thresholds of severity of dependence based on the AUDIT score.

## Materials and Methods

### Sample

Included in the study of AAI in ED were 164 adult patients (122 men and 42 women) admitted to the 24-h ED of the Centre Hospitalier Universitaire (CHU) Gabriel Montpied in Clermont Ferrand, France, over a 2-month period in 2008. Patients were included as and when their admission and we did not made calculation of population. The experimental protocol had previously been approved by the Committee for the protection of individuals [Comité de Protection des Personnes (CPP)]. The inclusion criteria were diagnosis of alcohol acute intoxication as the principal diagnosis or an additional diagnosis [DSM-IV criteria, Ref. (36)] and a blood alcohol level (BAL) >0.8 g/L, at the time of ED admission. Of those eligible during this 2-month period 6 refused to participate in the study, 17 had serious medical conditions which precluded their participation, and 4 were not included for other reasons, representing an 86% participation rate. Informed consent was obtained after the patient had reached a zero BAL, when his mental state made a psychometric evaluation possible. Following signed informed consent patients were interviewed regarding socio demographic characteristics, medical history, and clinical and psychometric measures.

### Data collection and instruments

Interviews were conducted in a private area of the ED to maintain confidentiality by trained interviewers (Julie Geneste, Benjamin Arnaud, Georges Brousse) using a structured interview schedule that averaged about 50 min in length. Participants were given French versions of the screening instruments for problem drinking (CAGE, RAPS4-QF, and AUDIT). The CAGE questionnaire was developed to detect life time alcohol dependence (26); (1) Have you ever felt you should *C*ut down on your drinking? (2) Have people *A*nnoyed you about your drinking? (3) Have you ever felt bad or *G*uilty about your drinking? (4) Have you ever had a drink first thing in the morning to steady your nerves or get rid of a hangover (*E*ye opener)? Two or more positive answers are a common cut point for detecting alcoholism (26, 37). The AUDIT was developed by the World Health Organization (38) to identify problem drinkers in primary care settings. This 10-item scale includes questions to assess alcohol intake, alcohol dependence, and alcohol-related problems. The French version of the AUDIT showed good discrimination for dependent patients with a cut point of 13 or hazardous drinkers with a cut point of 7 in the general population (32). The rapid alcohol problem screen (RAPS) was developed by Cherpitel (39), to detect current alcohol dependence and consists of the following four items: (1) During the last year, have you had a feeling of guilt or remorse after drinking? (*R*emorse), (2) During the last year, has a friend or family member ever told you about things you said or did while you were drinking that you could not remember? (*A*mnesia, also called Blackouts), (3) During the last year, have you failed to do what was normally expected from you because of drinking? (*P*erform), (4) Do you sometimes take a drink in the morning, when you first get up? (*S*tarter, also called eye opener). The RAPS4-QF includes the RAPS4 items plus two additional questions: (a) During the last year, have you had five or more drinks on at least one occasion? (*Q*uantity), (b) During the last year, do you drink as often as once a month? (*F*requency). A positive response on any one of the four RAPS4 items or both of the quantity–frequency items is considered positive on the RAPS4-QF for alcohol abuse or dependence (29). The RAPS4 and RAPS4-QF were translated into French using the well-recognized forward–backward translation technique (40). In order to investigate an optimal cut point for RAPS4-QF a series of scores corresponding to different cut points on an incremental scale (as for the other scales) was used [for more detail on the screening test used see Ref. (35)]. For all screening instruments, participants were questioned about the last 12 months. Alcohol dependence was established from a positive response in three or more of the seven domains on DSM-IV diagnostic criteria as measured by the Alcohol Section of the French version of the mini international neuropsychiatric interview 5.0.0 [MINI, Ref. (41)], while harmful drinking/abuse was established from a positive response on one or more of the four consequence items related to abuse on the DSM-IV.

### Data analysis

SPSS software version 15.0 was used for statistical analysis. In the previous study, means were compared using parametric (Student’s *t*-test, ANOVA) or non-parametric tests (Mann–Whitney for non-normal distributions). Bonferroni corrections were applied to *t*-tests to reduce the likelihood of significant findings based on multiple comparisons. To investigate threshold scores (TSs) that optimized the sensitivity and specificity of the scales for detecting alcohol abuse or dependence, four measures were used: (i) Youden’s index, (ii) efficiency, (iii) receiver operating characteristic (ROC) curves technique, and (iv) quality ROC curve [QROC; (42)]. The choice of the optimized TS for each instrument was made by calculating the indices of quality (χ^2^) proposed by Kraemer (42) [for more detail see Ref. (35)].

For each optimal threshold score, the Predictive Value of a Positive test (PVP: proportion of those with a positive test also having a positive diagnosis), and the Predictive Value of a Negative test (PVN: proportion of those with a negative test also having a negative diagnosis) were calculated using Bayes’ theorem. Otherwise, to explore the relation between the AUDIT and the MINI and in order to propose thresholds of severity for the AUDIT, the sum of the positive items on the MINI (dependence) were analyzed (1) as a quantitative variable (Spearman correlation coefficient), and (2) as ordinal parameter (Kruskal–Wallis test followed by Dunn’s test), using two-sided tests with a type I error set at α = 0.05.

## Results

### Demographic and drinking characteristics

Among the 164 patients, 122 (74.40%) were male. The mean age of the sample was 46 years (SD = 11.6). About half of the participants lived alone (50%, *N* = 82), and a third were unemployed. A history of treatment for alcohol-related disorders was reported by 64.63% of the patients. No gender difference was obtained for demographic characteristics, except for employment status (with men more likely to be employed than women *p* = 0.007). Of the sample, 11.60% (*N* = 19) were diagnosed as alcohol abusers and 78.05% (*N* = 128) as alcohol dependent with the MINI [for more detail see Ref. (35)].

### Intervals of severity of AUD based on scales’ thresholds

**Figure 1:**
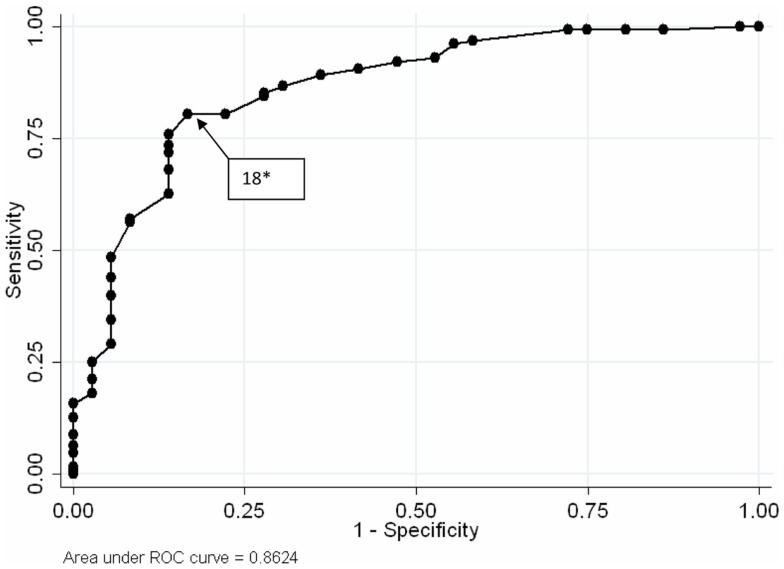
**Receiver operating characteristic curves AUDIT for patients admitted for drunkenness**. *Optimal threshold scores {by calculating the indices of quality [number total of subjects × *k*(1,0) × *k*(0,0) (42)]} for detecting alcohol dependence. According to the MINI (41), alcohol dependence was established from a positive response in three or more of the seven domains on DSM-IV diagnostic criteria of dependence.

From the results reported in the earlier paper concerning the optimal thresholds for detecting alcohol abuse and alcohol dependence we have distinguished intervals of severity. The optimal cut point for the RAPS4-QF was ≥2 for men (χ^2^ = 70.68, *p* < 0.001) and ≥4 for women (χ^2^ = 12.47, *p* < 0.001). In the case of CAGE, the optimal cut point was ≥3 for men (χ^2^ = 22.37, *p* < 0.01) and ≥2 for women (χ^2^ = 28.37, *p* < 0.001). The optimal cut point for the AUDIT was ≥12 for men (χ^2^ = 44.48, *p* < 0.001) and ≥7 for women (χ^2^ = 28.38, *p* = 0.001). For detecting alcohol dependence, the optimal cut point for the RAPS4-QF was ≥3 for men (χ^2^ = 30.44, *p* < 0.001) and ≥4 for women (χ^2^ = 13.59, *p* < 0.001). For the CAGE, a cut point ≥3 was found for both men (χ^2^ = 24.42, *p* < 0.001) and women (χ^2^ = 17.01, *p* < 0.001). For AUDIT, the optimal cut was ≥14 for men (χ^2^ = 32.52, *p* < 0.001) and ≥11 for women (χ^2^ = 21.00, *p* < 0.001) [35]. Moreover, 18 was the optimal cut point of the AUDIT for the total sample (χ^2^ = 51.31, *p* < 0.001) (Figure 1).

On these results, we distinguished AUD severity intervals (Alcohol-related disorders risk, abuse, and dependence) depending on the two optimal cut points found for the RAPS4-QF for men (scores 0–1, 2, 3–4), the two optimal cut points found for the CAGE for women (scores 0–1, 2, 3–4), and the two optimal cut points found for the AUDIT (respectively scores 0–11, 12–13, 14–40 for men and 0–6, 7–10, 11–40 for women).

A strong correlation between the AUDIT score and the number of positives items on the MINI (dependence) (*r* = 0.70) was found. Three statistically different groups (*p* < 0.05) were isolated with the AUDIT: one or two positive items on the MINI (mean AUDIT scores between 9.5 and 10.3), three or four positive items on the MINI (mean AUDIT scores between 17.3 and 19.4), and five, six, or seven positive items on the MINI (mean AUDIT scores between 26.2 and 30.9) (Table 1).

**Table 1:** **Mean scores of the AUDIT according to number of DSM-IV criteria**.

MINI (number of	*N*	AUDIT	SD	MED
positive items)		scores mean		
1	8	9.5*	3.34	10
2	7	10.3*	4.39	10
3	25	17.3**	7.34	18
4	25	19.4**	6.78	21
5	27	26.2***	5.66	26
6	30	28.3***	7.12	29.5
7	25	30.9***	5.94	31
Total	147	23.1	9.12	

*Spearman’s rho = 0.7001 (*p* < 0.001)*.

Difference between AUDIT scores statistically significant between * and **, * and ***, **and *** (*p* < 0.05).

On these results, we have distinguished dependence severity intervals (mild dependence, moderate dependence, and severe dependence) depending on the optimal cut points found for the AUDIT (respectively scores 14–17, 18–25, 26–40 for men and scores 11–17, 18–25, 26–40 for women).

### Adjusted interventions to the severity of AUD

Given findings here, we propose, as a perspective, to adapt alcohol interventions based on severity of alcohol-related disorders indicated by cut points of the scales (Table 2).

**Table 2:** **Proposal for the choice of interventions based on results of RAPS-QF, AUDIT, or CAGE**.

Patient admitted to emergency for drunkenness (blood alcohol level 0.8 g/L)						
Men		Diagnosis	Women		Interventions	
RAPS4-QF*	AUDIT		CAGE	AUDIT	What?	Who?
<2	<12	ARD risk	<2	<7	Information and advice	ED workers
≥2 PVP: 97%; PVN: 100%	≥12 PVP: 100%; PVN: 42%	Abuse	≥2 PVP: 95%; PVN: 100%	≥7 PVP: 95%; PVN: 100%	Brief alcohol intervention	ED workers
≥3 PVP: 90%; PVN: 60%	≥14 PVP: 93%; PVN: 55%	Mild dependence	≥3 PVP: 88%; PVN: 80%	≥11 PVP: 86%; PVN: 100%	Brief negotiation interview referral to treatment	ED workers trained to alcohol interventions
≥3	≥18 PVP: 94%; PVN: 50%	Moderate dependence	≥3	≥18 PVP: 96%; PVN: 65%	
	≥26	Severe dependence		≥26	Exhaustive motivational intervention referral to treatment	Trained alcohol health workers

PVP, the predictive value of a positive test (proportion of those with a positive test also having a positive diagnosis); PVN, the predictive value of a negative test (proportion of those with a negative test also having a negative diagnosis) calculated using Bayes’ theorem. RAPS-QF*, score by implementation; ARD, alcohol-related disorders; ED, emergency department.

## Discussion

The CAGE, RAPS4-QF, and AUDIT screening tests are those most frequently used in the ED to identify problems associated with alcohol use (29, 39, 43–45). However, these screening tests are typically used for general populations admitted to ED and there are no recommendations for interpreting results related to sensitivity and specificity by gender and severity of alcohol disorders for these instruments (46, 47). The results of these instruments could provide guidance for optimizing interventions in the ED because patient’s level of engagement with treatment will depend on the strategy adopted at the initial consultation and this strategy could depend on the severity of the patient’s problem with alcohol (16, 48–50). Tests used to screen for and distinguish different levels of alcohol-related disorders should have good sensitivity at the suggested cut points for each level of severity.

Earlier, we reported results showing that RAPS-QF test (incremental score) seem to possess better psychometric properties than the CAGE in men while the CAGE seems more adapted to female populations (35). This finding was also reported by Cherpitel and Bazargan (51). Furthermore, different thresholds are evident depending on the severity of alcohol-related disorders (abuse or dependence). The AUDIT demonstrates good performance for detecting alcohol abuse and alcohol dependence in male patients, but at a higher cut point [12] than the cut point traditionally used [8] (52). In women, the recommended cut point of seven (46) is confirmed here for identifying AUDs. For the purpose of distinguishing alcohol dependence from other conditions, the AUDIT displays good performance at the cut point of 14 in men, 11 in women, and 18 for the total sample. In the study population here of those admitted to the ED for drunkenness, compared with the general population of those admitted to the ED, the higher cut point for the AUDIT is in agreement with that reported by Conigrave et al. (52) who advised a cut point of 15 (Se: 0.73, Spe: 0.84) for patients whose admission to emergency care was associated with acute alcoholism. Further, analysis of optimal thresholds based on the indices of quality for the AUDIT suggest the possibility of defining different cut points depending on the seriousness of the alcohol-related disorder beyond the classical distinction between abuse and dependence.

Thus our results demonstrate three severity intervals, depending on the two optimal points found for the RAPS4-QF for men and the two optimal cut points found for the CAGE for women. Similarly, our study demonstrate severity intervals depending on the cut point on the AUDIT (scores 0–11, 12–13, 14–40 for men and 0–6, 7–10, 11–40 for women). These cut points are compatible with risk intervals recently suggested by Rubinsky et al. (47) (0–4, 5–10, 11–14, 15–40 for men and 0–1, 2, 3–4, 5–8, 9–12, 13–40 for women), which could encompass, respectively, zones of occasional use (I), risky use and abuse (II), and dependence (III, scores 16–19; and IV scores 20–40) according to the World Health Organization (WHO) guidelines for the AUDIT (53). In this paper, risk intervals III (16–19) and IV (20–40) suggest a possible approach of the severity of dependence with the AUDIT scores.

The different thresholds proposed for CAGE, RAPS4-QF, and AUDIT could be used to guide the choices of intervention for the patient. Two “diagnoses tracking and interventions” can be proposed: a short diagnosis track using CAGE and RAPS4-QF, or a long diagnosis track using AUDIT. Thus, if one considers AUDIT too time consuming for persons admitted to the ED, the severity intervals could be designated in men and in women using, respectively, the RAPS-QF and the CAGE scales, because of their good performance in these populations. The score could help practitioners choose appropriate interventions, depending on the severity of alcohol-related disorders. Bazargan-Hejazi et al. (54) in a study testing the effect of brief interventions in the ED have underlined the efficacy of brief intervention for patients screened positive for at-risk drinking as defined by AUDIT scores of 7–18. Brief interventions were not effective for patients with scores in the 19–40 range, which could refer in this study to dependence. These results are compatible with a recent review published by Saitz (16) who highlighted the absence of evidence for efficacy of brief interventions in primary care in people with dependence or very heavy drinking. For these patients more lengthy MI, should be proposed (50). It requires being able to distinguish mild and moderate dependence (which requires BNI) to severe dependence (which requires MI) taking account gender. Consequently, it is necessary to screen patients in order to provide the most efficient intervention. Thus for patients admitted to ED for AAI we proposed, tacking account gender, the use of the AUDIT or CAGE, and the RAPS4-QF for screening with cut points orienting the choice of interventions. Interventions would be provided by ED workers (nurses or physicians) sensitized to brief interventions when screening tests guide toward alcohol abuse (12 ≤ AUDIT < 14 for men or 7 ≤ AUDIT < 11 for women or 2 ≤ RAPS4-QF < 3 for men or 2 ≤ CAGE < 3 for women) and by trained staff when screening tests guide toward very heavy drinking or dependence (14 ≤ AUDIT for men or 11 ≤ AUDIT for women, or 3 ≤ RAPS4-QF for men or 3 ≤ CAGE for women).

Moreover literature and experience suggest it would be important to propose more adapted interventions appropriate to the severity of dependence, since the more severe the disorder the stronger the denial and resistance to treatment. Additional analysis of thresholds for the AUDIT from the sum of items of the MINI allowed us to distinguish three supplementary severity intervals: a zone of mild dependence (AUDIT score 14–17 for men, 11–17 for women), a zone for moderate dependence (AUDIT score 18–25), and a zone of severe dependence (AUDIT score ≥26). For these thresholds, we propose adapted interventions that can use the brief negotiation approach for mild and moderate dependence and longer more intensive MI (more time consuming, requiring trained alcohol health workers) for severe dependence. These types of interventions require a thorough understanding of problematic alcohol use and solid training in this approach for nurses or practitioners who provide it (55).

Several limitations apply, however to our results. First, only the AUDIT provides threshold for levels of dependence severity. Short questionnaires like the CAGE and RAPS4-QF are not sufficient for this distinction in order to adapt accordingly the type of intervention. Indeed the AUDIT, for patients admitted for AAI, seems the more efficient screening test. However, the CAGE and RAPS4-QF can be used in ED setting where time required for AUDIT cannot be given. Secondly, we should have correlated our results with the recent definition of alcohol use disorder [DSM5, Ref. (56)]. Nevertheless, patients were not initially evaluated with this classification, which was not the gold standard diagnosis when we performed our work. However, we can assume that the levels of current severity proposed by the DSM5 for Moderate (presence of four to five symptoms) and Severe (presence of six or more symptoms) could correspond to a higher AUDIT score to 18. Further studies may confirm this. In the same way, the use of the MINI according to a uni-dimensional perspective can be criticized, however, this approach is the one retained in the new version of the DSM5. Finally, it is difficult to propose intervention according to a criterion of severity of AUDs defined by this only study. In fact it should have done a follow-up study to allow recommendations for different interventions and a control population. Today, review of the literature on brief interventions is challenged and our ambition was to offer more intervention strategies that determine the best intervention. Naturally, we followed the clinical intuition that leads us to think that interventions must be more intense when clinicians faced with a severe disorder, but this is not demonstrated with this study. It is also not known whether the level of denial and resistance to treatment depends only on severity of dependence or whether it may be associated with other factors such as readiness to change, independent of the level of severity of AUD (57). Undoubtedly, studies should be conducted in order to validate the relevance of the guidelines proposed here.

## Conclusion

Patients with alcohol-related disorders are frequent in the ED. Among them those admitted following acute intoxication represent a specific population. They are frequently alcohol dependent. During their presence in the ED they can benefit from intervention for an alcohol-related disorder. ED practitioners have to decide quickly who can benefit most from an intervention and the nature of the intervention. The choice is guided by knowledge of the severity of the disorder and by reserving lengthy and specialized interventions applied by highly trained caregivers when the severity of AUD suggests a graduated motivational approach. The systematic use of the CAGE, RAPS4-QF, or AUDIT in order to screen for the severity of alcohol-related disorders should be recommended in this setting.

## Conflict of Interest Statement

The authors declare that the research was conducted in the absence of any commercial or financial relationships that could be construed as a potential conflict of interest.
